# Editorial: The Unusual Suspects: Linguistic Deficits in Non-Language-Dominant Neurodegenerative Diseases

**DOI:** 10.3389/fnagi.2022.861041

**Published:** 2022-02-16

**Authors:** Adolfo M. García, Agustín Ibáñez, Bruce Miller, Maria Luisa Gorno Tempini

**Affiliations:** ^1^Cognitive Neuroscience Center, Universidad de San Andrés, Buenos Aires, Argentina; ^2^Global Brain Health Institute, University of California, San Francisco, San Francisco, CA, United States; and Trinity College Dublin, Dublin, Ireland; ^3^National Scientific and Technical Research Council (CONICET), Buenos Aires, Argentina; ^4^Departamento de Lingüística y Literatura, Facultad de Humanidades, Universidad de Santiago de Chile, Santiago, Chile; ^5^Latin American Brain Health Institute (BrainLat), Universidad Adolfo Ibáñez, Santiago, Chile; ^6^Department of Neurology, Memory and Aging Center, University of California, San Francisco, San Francisco, CA, United States

**Keywords:** speech, language, Alzheimer's disease, mild cognitive impairment, frontotemporal lobar degeneration, Parkinson's disease

Discussions on linguistic deficits in neurodegenerative diseases are often circumscribed to primary progressive aphasia. Yet, verbal dysfunctions are also pervasive across neurodegenerative diseases typified by mnesic, socio-cognitive, or motoric alterations (García et al., 2022). This has been shown, for instance, in Alzheimer's disease (AD) (Taler and Phillips, 2008), behavioral variant frontotemporal dementia (bvFTD) (Geraudie et al., 2021a,b)(Geraudie et al., 2021a,b), progressive supranuclear palsy syndrome (PSPs) (Peterson et al., 2021), corticobasal syndrome (CBS) (Peterson et al., 2021), and Parkinson's disease (PD) (Birba et al., 2017). With a few exceptions (Boschi et al., 2017; García et al., 2022), however, relevant evidence has been compiled for each disorder separately, failing to foreground the transnosological import of language assessments in behavioral neurology. The present Research Topic directly addresses this need.

We bring together ten articles examining language difficulties in the abovementioned conditions. The evidence spans diverse linguistic dimensions (cutting across phonological, lexico-semantic, syntactic, and discursive-pragmatic levels), language families (Germanic, Indo-Aryan, Romance, Uralic), and methods (standardized batteries, experimental tasks, and spontaneous discourse analysis, in some cases combined with neuroimaging measures). Contributions are organized in three sets, dealing with (i) AD and mild cognitive impairment (MCI), (ii) frontotemporal lobar degeneration syndromes, and (iii) PD.

Opening the first set, Kaskikallio et al. examined neural correlates of verbal fluency in Finnish speakers with either AD or MCI alongside healthy participants. Behavioral outcomes were associated with white matter hyperintensities in bilateral fronto-parieto-occipital as well as right temporal regions, suggesting that vocabulary search difficulties involve cross-lobar axonal disruptions. The second report, by Itaguchi et al., zoomed into animal fluency in Spanish-speaking AD patients. Relative to controls, these patients exhibited more intrusions at the start of the task and more perseverations toward the end. Patients with high error rates presented with marked alterations along left frontal tracts, reinforcing the importance of white matter integrity for fluency performance. Moving onto the textual domain, Bose et al. analyzed aspects of connected speech in Bengali speakers with AD. In addition to reduced speech rate, semantic richness, and sentential complexity, patients exhibited fewer pronouns—the opposite of what is typically reported in English speakers. This observation invites much-needed comparisons between well-documented and under-researched languages. For their part, Maziero et al. assessed textual inference skills in Portuguese speakers with MCI. Deficits were observed in subgroups with amnestic and non-amnestic profiles, best predicted by verbal memory in the former and semantic knowledge in the latter. These results suggest that pragmatic skills may be affected in persons at increased risk for AD and associated with diverse components of declarative memory.

The second set deals with frontotemporal lobar degeneration. Berthier et al. report on two Spanish speakers with PSPs and echolalic dynamic aphasia. Verbal production deficits and echolalic behaviors (including echoing approval) were observed alongside inhibitory, socio-cognitive, and psychiatric alterations. Both patients presented with atrophy of the midbrain tegmentum and the superior medial frontal cortex. The authors surmise that abnormalities in these regions would involve inhibitory deficiencies compromising language control. Additional insights are provided by Peterson et al., who assessed general language skills in English speakers with PSPs and CBS. Both groups exhibited similar deficits across subtests of motor speech as well as phonological, semantic, and syntactic skills. Though less severe, these impairments resembled those of patients with non-fluent variant primary progressive aphasia. Impairments were associated with left frontal, striatal, and temporal abnormalities, suggesting shared neurolinguistic patterns across the three groups. Finally, Ruiz-Garcia et al. compared semantic and grammatical features of sentence production in English speakers with bvFTD and AD. The former group wrote longer sentences, more often addressed to the examiner and focused on interpersonal relationships. Such difficulties were associated with general cognitive status in AD, but not in bvFTD. Thus, overlooked sentential features might inform differential characterizations in these populations.

The section on PD opens with a study on semantic memory and lexical availability in Spanish (Cardona et al.). The authors observed impaired naming (in response to pictorial and verbal cues) and impoverished lexical access in larger and smaller semantic fields. Difficulties were prominent for non-living entities, yielding high classification between patients and controls. Results are interpreted as a disruption of categorization skills and embodied mechanisms. Embodied considerations also figure prominently in Baez et al. study on Spanish speakers with PD. Two sentence-level tasks revealed difficulties in specific syntactic functions (functional-role assignment) and socio-emotional dimensions (*Schadenfreude*), irrespective of overall cognitive and affective status. Classification between patients and controls was improved when these measures were considered jointly, highlighting the usefulness of multidimensional language assessments in the disease. The relevance of embodied approaches to PD is further emphasized by Gianelli et al. Their mini-review compiles evidence from action fluency and action naming studies revealing partly selective deficits in early-stage patients. Action-semantic tasks are thus proposed as a complement to standard clinical assessments and interventions in PD.

Collectively, these articles illustrate the multilevel, cross-linguistic, and transnosological importance of linguistic assessments in non-primarily linguistic neurodegenerative diseases. Systematic speech and language evaluations can promote fine-grained characterizations of each disorder, inform neurocognitive models, and even nurture the quest for transdiagnostic and disease-specific markers –a most pressing task given the escalating growth of neurodegeneration worldwide. May this Research Topic inspire future work in the same direction.

## Author Contributions

AG wrote the manuscript. AI, BM, and MG revised the manuscript. All authors approved submission of the manuscript.

## Funding

This work was supported by CONICET and FONCYT-PICT (Grant Nos. 2017-1818 and 2017-1820). AG is a Atlantic Fellow at the Global Brain Health Institute (GBHI) and was supported with funding from GBHI, Alzheimer's Association, and Alzheimer's Society (Alzheimer's Association GBHI ALZ UK-22-865742); ANID, FONDECYT Regular (1210176); Programa Interdisciplinario de Investigación Experimental en Comunicación y Cognición (PIIECC), Facultad de Humanidades, USACH. AI was supported by grants of Alzheimer's Association GBHI ALZ UK-20-639295, Takeda CW2680521, ANID/FONDECYT Regular (1210195); ANID/FONDAP 15150012, Sistema General de Regalías (BPIN2018000100059), Universidad del Valle (CI 5316), and the Multi-Partner Consortium to Expand Dementia Research in Latin America (ReDLat), funded by the National Institutes of Aging (NIA) of the National Institutes of Health (NIH) under award number R01AG057234, an Alzheimer's Association grant (SG-20-725707-ReDLat), the Rainwater Foundation, and the Global Brain Health Institute. MG was supported by grants from the National Institutes of Health (NINDS R01 NS050915, NIDCD K24 DC015544, and NIA U01 AG052943).

## Author Disclaimer

The content is solely the responsibility of the authors and does not represent the official views of the National Institutes of Health, Alzheimer's Association, Rainwater Charitable Foundation, or Global Brain Health Institute.

## Conflict of Interest

The authors declare that the research was conducted in the absence of any commercial or financial relationships that could be construed as a potential conflict of interest.

## Publisher's Note

All claims expressed in this article are solely those of the authors and do not necessarily represent those of their affiliated organizations, or those of the publisher, the editors and the reviewers. Any product that may be evaluated in this article, or claim that may be made by its manufacturer, is not guaranteed or endorsed by the publisher.
